# Oral lesions in Tuberculosis

**DOI:** 10.11604/pamj.2015.22.336.8420

**Published:** 2015-12-04

**Authors:** Sandhya Gokavarapu, Prashanth Panta

**Affiliations:** 1 Basavatarakam Indo Americal Cancer Hospital and Research Institute, Banjara Hills, Hyderabad, India; 2 Department of Oral Medicine and Radiology, MNR Dental College and Hospital, Narsapur road, Sangareddy (502294), Medak District, Telangana, India

**Keywords:** Oral tuberculous ulcer, atypical oral lesions, oral cancer

## Image in medicine

A 50 year old male was referred to a tertiary cancer hospital. On inquiry, the patient revealed a painful, non healing ulcer on the cheek, since one month. He was a habitual smoker; his medical history was non-significant and the neck was negative for lymph nodes. Clinical examination revealed a diffuse ulcero-indurative lesion extending from the left lip commissure to the left buccal mucosa (A and B). A biopsy was performed at the buccal mucosa and the findings confirmed the absence of malignancy, but it rather described granulomatous tissue admixed with multinucleated foreign body gaint cells and inflammatory cell infiltrate. Considering the granulamatous nature, the lesion was rebiopsied and Ziehl Neelsen stain was applied; it clearly indicated a tuberculous etiology. Sputum was positive for Acid Fast Bacilli (10-99AFB/100 Field, grading 1+). Postero-anterior chest radiograph showed obliteration of right CP angle (pleural thickening), irregular fibrotic lesions and calcifications in both upper and middle lobes (sequelae of old koch's). Interestingly, this patient never had any symptoms of pulmonary tuberculosis. Though the incidence of oral lesions in tuberculosis is low, it should be included in the differential diagnosis of atypical oral lesions and oral squamous cell carcinoma.

**Figure 1:**
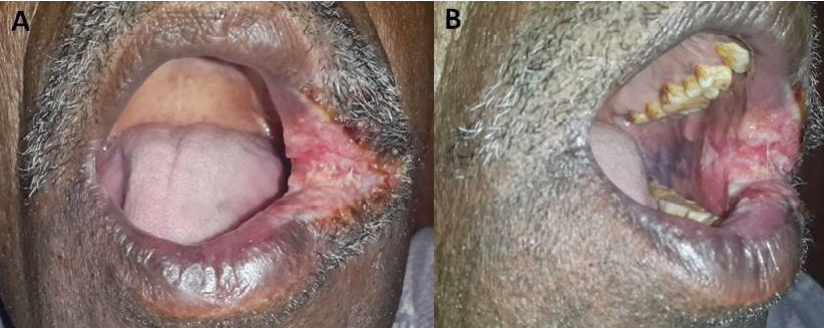
(A and B) diffuse ulcer involving the left lip commissure and buccal mucosa

